# Synthesis of Non-linear Protein Dimers through a Genetically Encoded Thiol-ene Reaction

**DOI:** 10.1371/journal.pone.0105467

**Published:** 2014-09-02

**Authors:** Jessica Torres-Kolbus, Chungjung Chou, Jihe Liu, Alexander Deiters

**Affiliations:** 1 Department of Chemistry, North Carolina State University, Raleigh, North Carolina, United States of America; 2 Department of Chemistry, University of Pittsburgh, Pittsburgh, Pennsylvania, United States of America; University of Pittsburgh, United States of America

## Abstract

Site-specific incorporation of bioorthogonal unnatural amino acids into proteins provides a useful tool for the installation of specific functionalities that will allow for the labeling of proteins with virtually any probe. We demonstrate the genetic encoding of a set of alkene lysines using the orthogonal PylRS/PylT_CUA_ pair in *Escherichia coli*. The installed double bond functionality was then applied in a photoinitiated thiol-ene reaction of the protein with a fluorescent thiol-bearing probe, as well as a cysteine residue of a second protein, showing the applicability of this approach in the formation of heterogeneous non-linear fused proteins.

## Introduction

Covalent attachment of proteins to ligands, polymers, and surfaces creates macromolecules combining specific biological function with favorable physical and chemical properties. For example, studying biological processes in their native environment often requires the addition of reporter tags to proteins [1]. To date, the mainstay tagging strategy for imaging of proteins involves genetic fusions of fluorescent proteins [2], [3]. However, the large size of fluorescent proteins can interfere with the folding and activity of the targeted protein [4]. Alternatively, tag-mediated labeling methods have been exploited, including self-labeling proteins, such as HaloTag, SNAP-tag, CLIP-tag, and enzyme-mediated labeling [5], [6]. Although these methods allow for smaller reporter tags, limitations with regard to the position and the structure of the label remain and the presence of an enzyme is required.

An alternative strategy to label proteins is via the introduction of a single-residue modification, which is nearly non-perturbing. Site-specific protein labeling can be achieved by the installation of tags through bioconjugation reactions with reactive handles previously installed in a protein by using an orthogonal aminoacyl-tRNA synthetase/aminoacyl-tRNA pair for unnatural amino acid (UAA) mutagenesis [7]–[10]. The bioorthogonal groups can be installed at virtually any position at the protein expressed in pro- and eukaryotic cells and the choice of probes is nearly limitless. Bioorthogonal chemical handles that have been genetically encoded for conjugation reactions include ketones [11]–[14], azides [15]–[20], alkenes [21]–[28], alkynes [24], [29]–[32], tetrazines [33], aryl halides [34], [35], and aryl boronates [36]. The alkene functionality is currently receiving considerable attention due to its versatility in organic transformations and it is rarely found in natural proteins [37], [38], allowing for selective modification. Carbon-carbon double bonds have been exploited for protein modification in reactions including olefin-metathesis [26], [39], photoaddition of tetrazoles [25], [27], [28], inverse electron demand Diels-Alder cycloadditions [23], [24], [30], and thiol-ene reactions [22], [40]–[44].

Bioorthogonal reactions have been applied in a variety of site-specific modifications of proteins such as fluorescent labeling, PEGylation, biotinylation, post-translational modification mimics, and surface immobilization [7], [9], [45]–[47]. Another area of interest for which bioconjugation reactions have been explored is in the generation of non-linear protein fusions. In biological systems, proteins often bind to other proteins to gain stability, affinity and higher specificity to perform specific cellular functions such as signal transduction, transcriptional regulation, and DNA repair [48]–[50]. Elucidation of many of these processes have led to the generation of chemical and biosynthetic methods to create non-linear protein linkages post-translationally for the control and performance of a number of functions, as well as protein trafficking and isolation. Methods that have been explored include native chemical ligation [51]–[54], enzyme based strategies [55],[56], and conjugation employing reactions with UAA residues [57]–[63]. The introduction of UAAs at a specific position allows for greater topological diversity with minimal protein modification [7]–[9], [46]. Here, we are applying the site-specific genetic incorporation of alkenes into proteins in the direct, spacer-free generation of non-linear protein fusions.

The thiol-ene reaction involves a radical-mediated addition of a thiol to an alkene that occurs upon UV irradiation (365–405 nm) [64], [65]. The reaction offers the possibility of using light to control both in space and time the formation of a stable thioether bond. As a result of its specificity for alkenes and compatibility with aqueous environments, the thiol-ene reaction is a bioorthogonal reaction that has been applied in polymer and material synthesis [66]–[72], carbohydrate modification [73], [74], and peptide and protein modification [21], [22], [29], [40]–[44]. Recently, orthogonal thiol-ene bioconjugations applying alkenyl UAAs and synthetic organic reaction partners have been reported [21], [22]. In order to expand the chemical diversity of these orthogonal handles, we demonstrate the synthesis, incorporation and protein heterodimer formation using alternative thiol-ene reaction conditions.

## Results and Discussion

### Incorporation of alkene lysines into proteins

**Figure 1:**
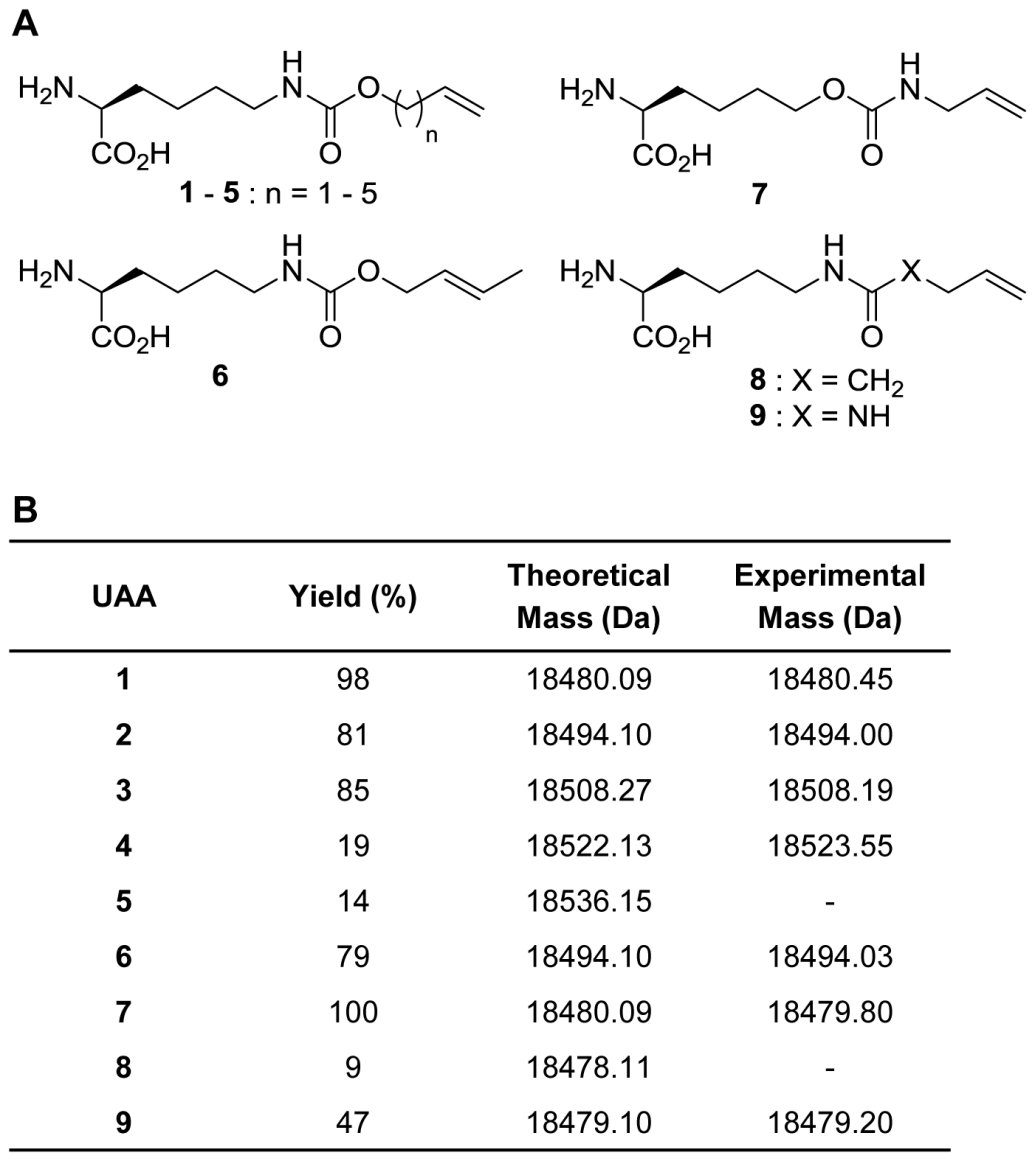
Genetic incorporation of alkene-lysine analogs into myoglobin by the wild-type *Mb*PylRS/PylT_CUA_ pair. (A) Structures of alkenyl lysine derivatives bearing an ε-carbamate linkage (**1**–**6**), an inverted carbamate **7**, an amide **8**, and an urea **9**. (B) Myoglobin comparative incorporation efficiencies (%) and ESI-MS results.

The pyrrolysyl-tRNA synthetase (PylRS), found in certain methanogenic archaea and bacteria, directly charges pyrrolysine (Pyl) onto its cognate tRNA that subsequently delivers it in response to an in-frame amber stop codon, TAG [75]–[77]. Furthermore, it has been demonstrated that the pyrrolysyl-tRNA synthetase/pyrrolysyl-tRNA_CUA_ pairs from *Methanosarcina barkeri* (*Mb*PylRS/PylT_CUA_) and *M. mazei* (*Mm*PylRS/PylT_CUA_) are functional in *Escherichia coli* [78], *Saccharomyces cerevisiae* [79], mammalian cells [80], *Caenorhabditis elegans* [81], [82], *Drosophila melanogaster* [83], and *Xenopus laevis* oocytes [84]. Furthermore, the wild-type PylRS is capable of accommodating a broad range of unnatural lysine derivatives based on a carbamate linkage at the ε-amino group to which a variety of functional groups, including *tert*-butyl [85], azido, alkynyl [17], norbornene [23], and diazirine [86] have been accommodated. We first synthesized a small collection of aliphatic alkene-lysines to diversify the structure of bioconjugation handles and to explore the ability to accommodate long-chain alkenes and lysine-linkages other than carbamates by the *Mb*PylRS (Figure 1A and Schemes S1–S4).

To investigate whether the synthesized alkene-lysines are substrates for the wild-type *Mb*PylRS, the incorporation efficiencies of **1**–**9** into myoglobin were evaluated by protein expression in *E. coli*. Cells were grown in the absence of an UAA and in the presence of **1**–**9**. The amino acids **1**, **2** and **3** have been previously described and incorporated into proteins using wild-type PylRS and/or PylRS mutants [22], [85]. Here we found that additional analogs can be efficiently incorporated into myoglobin by the *Mb*PylRS. The obtained incorporation efficiencies and ESI-MS results are listed in Figure 1B and the corresponding SDS-PAGE analysis is shown in Figure S1.

Previous crystallographic studies of PylRS have indicated that the synthetase holds a large hydrophobic pocket, capable of accommodating bulky and hydrophobic moieties [87], [88]. In addition, it has been found that the carbamate moiety at the lysine side-chain is an essential discriminator for substrate recognition. For instance, the oxygen atom adjacent to the side-chain carbonyl group in **1** interacts via a water-mediated hydrogen bond with the side-chain carbonyl group of Asn346, a key residue in establishing substrate recognition in PylRS [85], [87]. We found that the amino acid binding pocket of *Mb*PylRS exhibited flexibility to accommodate substrates **1**, **2**, **3**, **6** and **7** with amino acids **1** and **7** showing the highest incorporation efficiency, which could be explained by their smaller size. While the amino acids **4** and **5** were not efficiently incorporated into protein due to their longer carbon chains.

The successful incorporation of **1** and **7** into protein together with the inefficient substrate recognition of **8** and **9** by *Mb*PylRS suggests that the presence of an oxygen atom adjacent to the side-chain carbonyl group favors the hydrogen-bond network to be established more efficiently. We hypothesize that the recognition of **7** by *Mb*PylRS may be possible by re-directing the necessary interactions of the synthetase’s binding pocket to the *O* ^ε^-position. Moreover, we have previously observed a preference for the carbamate moiety over an amide group to drive the efficient genetic encoding of ε-*N*-propargyloxycarbonyl-lysine by the wild-type *Mb*PylRS/PylT_CUA_ pair, while its amide analog ε-*N*-pentynoyl-lysine was not accepted as a substrate [17]. Although analogs that bear a side-chain amide moiety have been incorporated into proteins by wild-type PylRS, so far only structures with up to four atom bonds in length from the amide ε-amino group have been tolerated by the enzyme’s binding pocket [89], [90]. Since our amino acid **8** is a bond longer, we can speculate that the carbamate functionality in **1**, compared to **8**, assists in an increase of substrate recognition efficiency by *Mb*PylRS, as the enzyme showed to also tolerate the lengthier amino acids **2** and **3**. The amino acid **9**, which bears a urea linkage, seemed to be slightly favored by *Mb*PylRS compared to the amide **8**. However, the amino acid **9** still proves to be a poor substrate compared to **1**. Our findings suggest that the replacement of the oxygen atom on the carbamate by a carbon or nitrogen atom may be enough to discriminate between the very similar substrates **1**, **8**, and **9**, possibly due to weaker interactions with the amide nitrogen atom or urea functionalities, thus not favoring an efficient binding of **8** or **9** into the *Mb*PylRS amino acid pocket.

**Figure 2:**
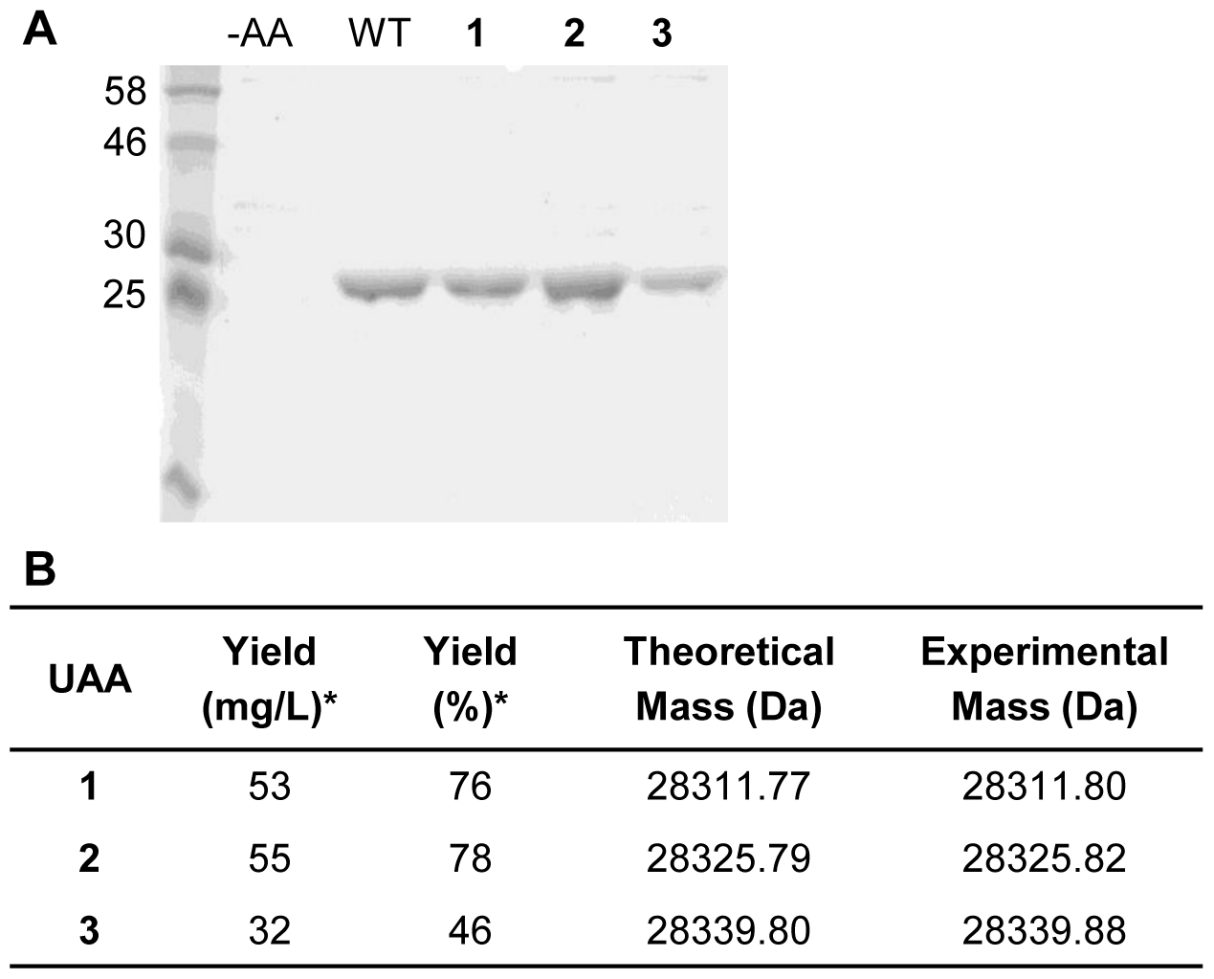
Genetic incorporation of alkene-lysine analogs 1, 2 and 3 into sfGFP. (A) SDS-PAGE analysis of purified sfGFP. –AA: no UAA was supplemented; WT: wild-type sfGFP; **1**, **2** and **3**: expression in the presence of the corresponding UAA (1 mM). (B) Protein yields (*wild-type sfGFP yield is 70 mg/L, 100%) and ESI-MS results.

With amino acids **1**–**3** showing good incorporation efficiency, we site-specifically incorporated these amino acids into superfolder Green Fluorescent Protein (sfGFP) as a second model protein in *E. coli*. We found that alkene lysines **1**–**3** were successfully introduced at position Y151 in sfGFP (Figure 2A) and that sfGFP yields were obtained at 32–70 mg/L, an approximately 10-fold increase of incorporation efficiency compared to myoglobin bearing the same amino acids **1**–**3**. ESI-MS analysis of purified sfGFP shows molecular weights corresponding to the site-specific incorporation of **1**, **2**, and **3** (Figure 2B).

### sfGFP labeling via the thiol-ene reaction

**Figure 3:**
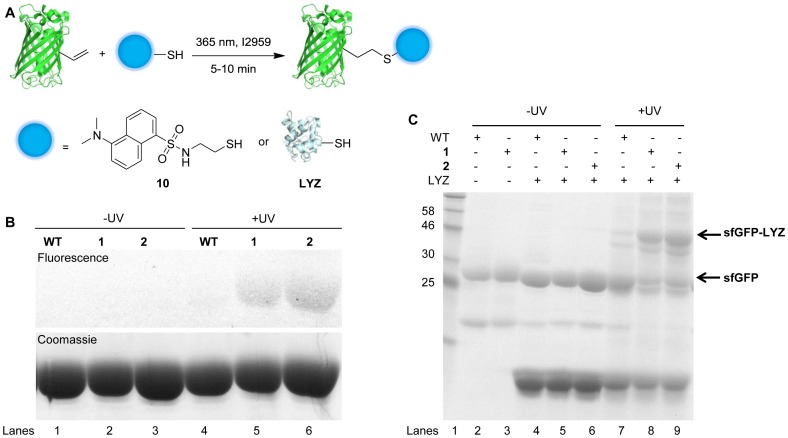
Alkenyl-sfGFP is fluorescently labeled with dansyl-thiol, and bioconjugated to lysozyme to assemble a non-linear protein dimer via the thiol-ene reaction. (A) sfGFP bearing an alkene functionality reacts photochemically with dansyl-thiol **(10)** or lysozyme (LYZ). (B) SDS-PAGE analysis demonstrates the labeling of alkenyl-sfGFP with **10** after 5 min of UV irradiation via thiol-ene ligation (lanes 5 and 6). Fluorescence (top) and Coomassie stain (bottom). (C) SDS-PAGE analysis shows mobility band shifts from 28 kD to 44 kD after samples were UV irradiated for 10 min (lanes 8 and 9), corresponding to the molecular weight of sfGFP-lysozyme conjugate. WT: wild-type sfGFP; **1** and **2**: sfGFP carrying the corresponding UAA; LYZ: lysozyme. –UV: samples were not exposed to UV irradiation. +UV: samples were irradiated at 365 nm for 5 or 10 min.

To verify that the thiol-ene reaction is suitable for labeling the alkene-bearing sfGFP, dansyl-thiol (**10**) was used as a fluorescent probe (Figure 3A and Scheme S5). Wild-type sfGFP and modified sfGFPs carrying **1** or **2**, which showed the highest incorporation efficiency, were subjected to a thiol-ene reaction with **10** by irradiating the reaction mixture with 365 nm UV light in the presence of the photoinitiator I2959 for 5 min. Both samples were then analyzed by SDS-PAGE gel and in-gel fluorescence imaging. Figure 3B shows that the alkene-containing sfGFPs modified with **1** and **2** were both selectively labeled with **10** after UV irradiation while the wild-type sfGFP was not fluorescently labeled. These results demonstrate that a thiol-containing fluorescence probe could be site-specifically conjugated to sfGFP bearing an alkene functional group.

In order to show the potential of the thiol-ene reaction in protein chemistry, we hypothesized that cysteine residues in another protein could also be used as a possible reaction partner, leading to the formation of a non-linear protein heterodimer (Figure 3A). Lysozyme is a small protein containing 8 cysteine residues within 129 amino acids [91]. The cysteines form 4 disulfide bonds and can be reduced to release free thiol groups. Analysis of bioconjugated proteins by SDS-PAGE revealed bands of expected molecular weight, as the bands corresponding to sfGFP increased from 28 kD to 44 kD via conjugation to lysozyme after UV exposure in the presence of the photoinitiator I2959 for 10 min (Figure 3C). This result indicates that the majority of the observed products are sfGFP-lysozyme heterodimers since lysozyme was supplied in 4 fold excess compared to alkenyl sfGFP. Without UV irradiation, no significant mobility shift was observed. As expected, wild-type sfGFP did not undergo a thiol-ene reaction with lysozyme. Overall, a successful protein-protein heterodimer formation via thiol-ene conjugation of an alkene-containing protein was achieved.

In both bioconjugation strategies we found that the addition of sodium dodecyl sulfate (SDS) was necessary for an efficient and specific conjugation reaction to alkene-labeled proteins within 5–10 min, in contrast to previously reported 1–2 h reaction times [22], [29], [43], thus significantly reducing UV exposure. We found that under our experimental conditions, lysozyme is (at least partially) denatured [92], [93], as confirmed by circular dichroism (CD) spectroscopy (Figure S2). This may result in more accessible cysteine residues and facilitate the thiol-ene bioconjugation reaction. Moreover, as a well-known surfactant, SDS has been proposed to form micelles in thiol-ene reactions for water-based polymerization reactions [94], [95]. It is possible that the association of the proteins with micelles may increase their local concentration, thus further facilitating the reaction.

## Conclusions

In conclusion, we have synthesized a collection of alkene lysines of varying length and ε-linkages and demonstrated their site-specific, genetically encoded incorporation into proteins in *E. coli* by the wild-type *Mb*PylRS/PylT_CUA_ pair. The alkene-containing amino acids **1**–**3** showed the highest incorporation efficiencies into myoglobin and protein yields decreased with increasing side-chain length, hinting the limitations of the wild-type synthetase’s binding pocket to accommodate sterically demanding amino acids. Among these amino acids, we also successfully incorporated the amino acid **7** with an inverted carbamate functionality at the ε-position of lysine. Replacement of the carbamate motif for an amide or urea failed to provide efficient incorporation into protein, once again suggesting that the carbamate moiety at the lysine side-chain can be an essential discriminator for substrate recognition by wild-type PylRS.

Next, the alkene amino acids **1**, **2**, and **3** were successfully incorporated into sfGFP, with **1** and **2** exhibiting the highest incorporation efficiency. Utilizing the thiol-ene reaction, alkene-bearing sfGFP was site-specifically bioconjugated to a dansyl-thiol fluorophore (**10**) upon irradiation with 365 nm of UV light in the presence of photoinitiator I2959 after only 5 min. In addition, we applied the site-specific genetic incorporation of alkene-bearing amino acids into proteins in the direct, spacer-free synthesis of a non-linear protein fusion of sfGFP and lysozyme. All components are recombinantly expressed and no post-translational introduction of functional groups was required. The work described herein demonstrates for the first time the assembly of a protein heterodimer by means of a light-induced thiol-ene ligation using genetically encoded alkene-bearing UAAs. This approach may become a promising tool to create non-linear proteins directly, with minimal synthetic effort, by creating direct protein-to-protein conjugations.

## Materials and Methods

### Synthesis of alkene lysines: general considerations

Unless otherwise stated, all reagents used were commercial reagents used without purification and reactions were performed under nitrogen using flame-dried glassware. The ^1^H NMR and ^13^C NMR spectra were recorded on a 300 MHz or 400 MHz Varian NMR spectrometer. The amino acid **1** was purchased from Chem-Impex International, Inc. For synthesis schemes of **2**–**10**, please refer to Schemes S1–S5.

### Procedure for the synthesis of 2a–5a

#### But-3-en-1-yl (2,5-dioxopyrrolidin-1-yl) carbonate (2a)

*N*,*N’*-Disuccinimidyl carbonate (353 mg, 1.38 mmol) was added to a solution of 3-buten-1-ol (60 µL, 0.69 mmol) and triethylamine (288 µL, 2.07 mmol) in dry acetonitrile (5 mL) at room temperature. The resulting mixture was stirred overnight and then concentrated under vacuum. The product was purified by column chromatography on SiO_2_ gel, eluted with 97∶2∶1 DCM/acetone/TEA to deliver **2a** (96 mg, 65%) as a colorless oil. ^1^H-NMR (400 MHz, CDCl_3_): δ 5.73 (m, 1 H), 5.15–5.08 (m, 2 H), 4.31 (t, *J* = 6.4 Hz, 2 H), 2.78 (s, 4 H), 2.46 (m, 2 H) ppm; ^13^C-NMR (100 MHz, CDCl_3_): δ 168.8, 151.5, 132.4, 118.4, 70.2, 32.7, 25.4 ppm.

#### 2,5-Dioxopyrrolidin-1-yl pent-4-en-1-yl carbonate (3a)

Compound **3a** (193 mg, 73%) was obtained as a colorless oil from 4-penten-1-ol (0.12 mL, 1.16 mmol) by following the procedure described above. ^1^H-NMR (400 MHz, CDCl_3_): δ 5.78 (m, 1 H), 5.09–5.01 (m, 2 H), 4.34 (t, *J* = 6.4 Hz, 2 H), 2.83 (s, 4 H), 2.17 (m, 2 H), 1.85 (m, 2 H) ppm; ^13^C-NMR (100 MHz, CDCl_3_): δ 168.6, 151.4, 136.5, 115.8, 70.6, 29.2, 27.3, 25.3 ppm.

#### 2,5-Dioxopyrrolidin-1-yl hex-5-en-1-yl carbonate (4a)

Compound **4a** (232 mg, 77%) was obtained as a colorless oil from 5-hexen-1-ol (0.15 mL, 1.25 mmol) by following the procedure described above. ^1^H-NMR (400 MHz, CDCl_3_): δ 5.73 (m, 1 H), 5.05–4.91 (m, 2 H), 4.27 (t, *J* = 6.4 Hz, 2 H), 2.77 (s, 4 H), 2.04 (m, 2 H), 1.70 (m, 2 H), 1.45 (m, 2 H) ppm; ^13^C-NMR (100 MHz, CDCl_3_): δ 169.2, 151.9, 138.2, 115.5, 71.7, 33.3, 28.0, 25.7, 24.9 ppm.

#### 2,5-Dioxopyrrolidin-1-yl hept-6-en-1-yl carbonate (5a)

Compound **5a** (202 mg, 67%) was obtained as a colorless oil from 6-hepten-1-ol (0.16 mL, 1.17 mmol) by following the procedure described above. ^1^H-NMR (400 MHz, CDCl_3_): δ 5.77 (m, 1 H), 5.00–4.91 (m, 2 H), 4.28 (t, *J* = 6.8 Hz, 2 H), 2.80 (s, 4 H), 2.05 (m, 2 H), 1.72 (m, 2 H), 1.39 (m, 4 H) ppm; ^13^C-NMR (100 MHz, CDCl_3_): δ 183.6, 168.9, 151.6, 138.5, 114.7, 71.6, 33.5, 28.3, 25.5, 24.9 ppm.

### Procedure for the synthesis of 2b–5b

#### (S)-6-(((But-3-en-1-yloxy)carbonyl)amino)-2-((tert-butoxycarbonyl)amino)hexanoic acid (2b)

Boc-L-Lys-OH (218 mg, 0.88 mmol) was added to a stirred solution of **2a** (157 mg, 0.74 mmol) in dry DMF (2 mL). The reaction was allowed to continue overnight at room temperature. The mixture was diluted in water (10 mL) and extracted with EtOAc (3×10 mL). The combined organic layers were washed with water (3×20 mL) and brine (10 mL). The resulting organic layer was dried over Na_2_SO_4_, filtered and concentrated in vacuo to dryness to furnish **2b** (219 mg, 86%) as an off-white foam. ^1^H-NMR (400 MHz, CDCl_3_): δ 11.15 (s, br, 1 H), 6.26 (s, br, 0.5 H), 5.75 (m, 1 H), 5.37 (m, br, 1 H), 5.09–5.01 (m, 2 H), 4.87, (s, br, 0.5 H), 4.26 (s, br, 1 H), 4.08 (m, br, 2 H), 3.13 (m, 2 H), 2.34 (m, 2 H), 1.81–1.40 (m, 15 H) ppm; ^13^C-NMR (100 MHz, CDCl_3_): δ 176.5, 157.2, 156.0, 138.5, 114.9, 80.1, 65.0, 53.3, 40.6, 33.5, 32.2, 29.5, 28.5, 22.5 ppm.

#### (S)-2-((tert-Butoxycarbonyl)amino)-6-(((pent-4-en-1-yloxy)carbonyl)amino)hexanoic acid (3b)

Compound **3b** (517 mg, 92%) was obtained as an off-white foam from **3a** (335 mg, 1.47 mmol) by following the procedure described above. ^1^H-NMR (300 MHz, CDCl_3_): δ 6.30 (m, br, 0. 5 H), 5.80 (m, 1 H), 5.27 (m, br, 1 H), 5.06–4.96 (m, 2 H), 4.80 (s, br, 0.5 H), 4.30 (s, br, 1 H), 4.06 (m, br, 2 H), 3.17 (m, 2 H), 2.10 (m, 2 H), 1.84–1.65 (m, 4 H), 1.58–1.35 (m, 13 H) ppm; ^13^C-NMR (100 MHz, CDCl_3_): δ 176.2, 157.1, 155.9, 137.7, 115.2, 80.3, 64.7, 53.5, 40.9, 32.5, 30.3, 29.7, 28.7, 22.8 ppm; HRMS-ESI (*m/z*): [M+K]^+^ calcd for C_17_H_30_N_2_O_6_ 397.1741, found 397.1726.

#### (S)-2-((tert-Butoxycarbonyl)amino)-6-(((hex-5-en-1-yloxy)carbonyl)amino)hexanoic acid (4b)

Compound **4b** (177 mg, 93%) was obtained as an off-white foam from **4a** (123 mg, 0.51 mmol) by following the procedure described above. ^1^H-NMR (400 MHz, CDCl_3_): δ 8.40 (s, br, 1 H), 6.29 (s, br, 0.5 H), 5.78 (m, 1 H), 5.31 (m, br, 1 H), 5.02–4.93 (m, 2 H), 4.90 (s, br, 0.5 H), 4.35 (s, br, 1 H), 4.05 (m, br, 2 H), 3.16 (m, 2 H), 2.06 (m, 2 H), 1.82–1.43 (m, 19 H) ppm; ^13^C-NMR (100 MHz, CDCl_3_): δ 176.3, 157.9, 155.7, 138.5, 114.8, 80.1, 64.9, 53.2, 40.6, 33.4, 32.1, 29.4, 28.5, 28.4, 25.2, 22.5 ppm; HRMS-ESI (*m/z*): [M+Na]^+^ calcd for C_18_H_32_N_2_O_6_ 395.2158, found 395.2145.

#### (S)-2-((tert-Butoxycarbonyl)amino)-6-(((hept-6-en-1-yloxy)carbonyl)amino)hexanoic acid (5b)

Compound **5b** (148 mg, 93%) was obtained as an off-white foam from **5a** (105 mg, 0.41 mmol) by following the procedure described above. ^1^H-NMR (300 MHz, CDCl_3_): δ 8.48 (s, br, 1 H), 6.33 (s, br, 0.5 H), 5.78 (m, 1 H), 5.30 (m, br, 1 H), 5.01–4.88 (m, 2.5 H), 4.29 (s, br, 1 H), 4.05 (m, br, 2 H), 3.16 (m, 2 H), 2.03 (m, 2 H), 1.78–1.18 (m, 21 H) ppm; ^13^C-NMR (75 MHz, CDCl_3_): δ 176.5, 157.3, 155.8, 138.9, 114.7, 80.3, 65.2, 53.3, 40.6, 33.8, 32.1, 29.6, 29.0, 28.7, 28.5, 25.5, 22.5 ppm; HRMS-ESI (*m/z*): [M+H]^+^ calcd for C_13_H_24_N_2_O_4_ 273.1809, found 273.1876.

### Procedure for the Boc-deprotection, 2–5

#### (S)-2-Amino-6-(((but-3-en-1-yloxy)carbonyl)amino)hexanoic acid HCl salt (2)

To a solution of **2b** (110 mg, 0.32 mmol) and Et_3_SiH (0.1 mL, 0.64 mmol) in dry DCM (4.5 mL), trifluoroacetic acid (0.24 mL, 3.2 mmol) was added dropwise, and the reaction mixture was allowed to stir at room temperature overnight. The volatiles were removed under reduced pressure and the residue was dissolved in a solution of 4 N HCl in 1,4-dioxane (0.25 mL) and DCM (0.75 mL), allowed to stir for 10 min at room temperature and then concentrated. The latter process was repeated two more times to ensure complete TFA to HCl salt exchange. The concentrated residue was dissolved in a minimal amount of MeOH and was precipitated into ice-cold Et_2_O. The precipitate was pelleted by centrifugation, the supernatant decanted, and the solid was washed with Et_2_O before drying under vacuum, affording the amino acid **2** (82.2 mg, 92%) as a white solid. ^1^H-NMR (400 MHz, DMSO-d6): δ 8.45 (s, br, 3 H), 7.09 (s, br, 1 H), 5.75 (m, 1 H), 5.10–5.01 (m, 2 H), 3.94 (t, *J* = 6.8 Hz, 2 H), 3.77 (t, *J* = 6.4 Hz, 1 H), 2.92 (m, 2 H), 2.23 (m, 2 H), 1.75 (m, 2 H), 1.36–1.26 (m, br, 4 H) ppm; ^13^C-NMR (100 MHz, DMSO-d6): δ 170.9, 156.2, 134.8, 117.0, 62.7, 51.8, 33.2, 29.6, 28.9, 21.6 ppm; HRMS-ESI (*m/z*): [M+H]^+^ calcd for C_11_H_20_N_2_O_4_ 245.1496, found 245.1490.

#### (S)-2-Amino-6-(((pent-4-en-1-yloxy)carbonyl)amino)hexanoic acid HCl salt (3)

Deprotection of **3b** (0.5 g, 1.39 mmol) was performed as described above to obtain **3** (0.40 g, 97%) as a white solid. ^1^H-NMR (400 MHz, D_2_O): δ 5.86 (m, 1 H), 5.07–4.98 (m, 2 H), 4.05–4.00 (m, 3 H), 3.11 (t, *J* = 5.2 Hz, 2 H), 2.10 (m, 2 H), 1.97–1.87 (m, 2 H), 1.69 (t, *J* = 6.4 Hz, 2 H), 1.56–1.39 (m, 4 H) ppm; ^13^C-NMR (75 MHz, D_2_O): δ 172.0, 158.9, 138.6, 114.9, 65.0, 52.7, 39.9, 29.6, 29.4, 28.5, 27.5, 21.6 ppm; HRMS-ESI (*m/z*): [M+H]^+^ calcd for C_12_H_22_N_2_O_4_ 259.1652, found 259.1653.

#### (S)-2-Amino-6-(((hex-5-en-1-yloxy)carbonyl)amino)hexanoic acid HCl salt (4)

Deprotection of **4b** (145 mg, 0.39 mmol) was performed as described above to obtain **4** (108.4 mg, 90%) as a white solid. ^1^H-NMR (400 MHz, DMSO-d6): δ 8.28 (s, br, 3 H), 7.08 (s, br, 1 H), 5.79 (m, 1 H), 5.02–4.93 (m, 2 H), 3.91 (t, *J* = 6.8 Hz, 2 H), 3.67 (s, br, 2 H), 2.94 (m, 2 H), 2.03 (m, 2 H), 1.76 (m, br, 2 H), 1.52 (t, *J* = 6.8 Hz, 2 H), 1.38 (m, br, 6 H) ppm; ^13^C-NMR (100 MHz, DMSO-d6): δ 171.1, 156.3, 138.5, 115.0, 63.4, 52.3, 32.8, 29.8, 29.0, 28.2, 24.6, 21.7 ppm; HRMS-ESI (*m/z*): [M+H]^+^ calcd for C_13_H_24_N_2_O_4_ 273.1809, found 273.1803.

#### (S)-2-Amino-6-(((hept-6-en-1-yloxy)carbonyl)amino)hexanoic acid HCl salt (5)

Deprotection of **5b** (130 mg, 0.336 mmol) was performed as described above to obtain **5** (104.4 mg, 96%) as a white solid. ^1^H-NMR (400 MHz, DMSO-d6): δ 8.45 (s, br, 3 H), 7.09 (m, br, 1 H), 5.78 (m, 1 H), 5.01–4.92 (m, 2 H), 3.90 (t, *J* = 6.4 Hz, 2 H), 3.82 (s, br, 1 H), 2.93 (m, 2 H), 2.01 (m, 2 H), 1.77 (m, br, 2 H), 1.52 (m, 2 H), 1.45–1.28 (m, 10 H) ppm; ^13^C-NMR (100 MHz, DMSO-d6): δ 171.0, 156.3, 138.7, 114.8, 63.5, 51.8, 33.1, 29.6, 28.9, 28.6, 28.0, 24.9, 21.6 ppm; HRMS-ESI (*m/z*): [M+H]^+^ calcd for C_14_H_26_N_2_O_4_ 287.1965, found 287.1957.

### Procedure for the synthesis of 6

#### (S)-6-(((But-2-en-1-yloxy)carbonyl)amino)-2-((tert-butoxycarbonyl)amino)hexanoic acid (6b)

Diphosgene (0.26 mL, 2.16 mmol) was added dropwise to an ice-cold mixture of 2-buten-1-ol (*cis*:*trans* isomers, ∼1∶19) (0.12 mL, 1.66 mmol) and potassium carbonate (0.69 g, 4.98 mmol) in dry Et_2_O (5 mL). The resulting mixture was allowed to stir overnight at room temperature, filtered and carefully concentrated under reduced pressure to avoid loss of the volatile product. The chloroformate **6a** was obtained as a clear liquid and without further purification it was dissolved in THF (1 mL). Then, it was added dropwise to an ice-cold solution of Boc-L-Lys-OH (495 mg, 2.0 mmol) in 1 M NaOH aqueous (1 mL) and THF (4 mL). The reaction was allowed to run overnight at room temperature. The volatiles were removed under reduced pressure and the residue was diluted in water and then washed with EtOAc (10 mL). The water layer was acidified with 5% citric acid to pH 3–4 and extracted with EtOAc (3×10 mL). The combined organic layers were washed with water (20 mL) and brine (10 mL). The resulting organic layer was dried over Na_2_SO_4_, filtered and concentrated in vacuo to dryness to furnish **6b** (343 mg, 60%) as an off-white foam. ^1^H-NMR (400 MHz, CDCl_3_): δ 8.40 (s, br, 1 H), 6.29 (s, br, 0.5 H), 5.78 (m, 1 H), 5.31 (m, br, 1 H), 5.02–4.90 (m, 2.5 H), 4.29 (s, br, 1 H), 4.05 (m, br, 2 H), 3.15 (m, 2 H), 2.07 (m, 2 H), 1.81–1.40 (m, 15 H) ppm; ^13^C-NMR (75 MHz, CDCl_3_): δ 176.4, 156.9, 156.0, 131.0, 125.9, 80.1, 65.7, 53.3, 40.6, 32.2, 29.5, 28.5, 22.5, 17.9 ppm; HRMS-ESI (*m/z*): [M-H]^−^ calcd for C_16_H_28_N_2_O_6_ 343.1864, found 343.1869.

#### (S)-2-Amino-6-(((but-2-en-1-yloxy)carbonyl)amino)hexanoic acid TFA salt (6)

To a solution of **6b** (317 mg, 0.92 mmol) and Et_3_SiH (0.29 mL, 1.84 mmol) in dry DCM (13 mL), trifluoroacetic acid (0.68 mL, 9.20 mmol) was added dropwise, and the reaction mixture was allowed to stir at room temperature overnight. The volatiles were removed under reduced pressure and the residue was dissolved in a minimal amount of MeOH and precipitated into ice-cold Et_2_O. The precipitate was pelleted, the supernatant decanted, and the solid was washed with Et_2_O before drying under vacuum, affording the amino acid **6** (288 mg, 87%) as a white solid. ^1^H-NMR (400 MHz, D_2_O): δ 5.80 (m, 1 H), 5.58 (m, 1 H), 4.43 (d, *J* = 5.6, 2 H), 3.85 (m, 1 H), 3.08 (t, *J* = 6.0 Hz, 2 H), 1.88 (t, *J* = 6.0 Hz, 2 H), 1.66 (d, *J* = 6.4, 3 H), 1.53–1.33 (m, 4 H) ppm; ^13^C-NMR (100 MHz, DMSO-d6): δ 171.3, 156.1, 129.6, 126.6, 64.1, 52.7, 38.4, 30.1, 29.1, 26.5, 21.9, 21.6, 17.5 ppm; HRMS-ESI (*m/z*): [M+H]^+^ calcd for C_11_H_20_N_2_O_4_ 245.14958, found 245.14970.

### Procedure for the synthesis of 7

#### (S)-6-((Allylcarbamoyl)oxy)-2-((tert-butoxycarbonyl)amino)hexanoic acid (7a)

6-Hydroxy-Boc-L-norleucine-OH (25 mg, 0.10 mmol) was dissolved in a solution of dry DCM (1 mL) and DIPEA (53 µL, 0.30 mmol). The solution was chilled to 0°C before the addition of allyl isocyanate (18 µL, 0.20 mmol) and the reaction was allowed to proceed at 40°C overnight. After cooling to room temperature, the mixture was diluted with DCM (3 mL) and 5% citric acid (4 mL) was added. The aqueous layer was extracted with DCM (3×4 mL) and the combined organic layers were washed with water (10 mL) and brine (5 mL). The resulting organic layer was dried over Na_2_SO_4_, filtered and concentrated in vacuo to dryness to furnish **7a** (29 mg, 89% yield) as an off-white foam. ^1^H-NMR (400 MHz, CDCl_3_): δ 5.85 (m, 1 H), 5.24–5.07 (m, 2 H), 4.74 (m, br, 1 H), 4.29, (s, br, 1 H), 4.06 (t, *J* = 5.6 Hz, 2 H), 3.78 (m, 2 H), 1.93–1.25 (m, 15) ppm; ^13^C-NMR (100 MHz, CDCl_3_): δ 176.0, 155.8, 135.0, 116.4, 80.3, 64.8, 53.3, 43.3, 32.2, 28.7, 28.5, 22.0 ppm; HRMS-ESI (*m/z*): [M+Na]^+^ calcd for C_15_H_26_N_2_O_6_ 353.1689, found 353.1654.

#### (S)-6-((Allylcarbamoyl)oxy)-2-aminohexanoic acid TFA salt (7)

Deprotection of **7a** (28 mg, 0.085 mmol) was performed by following the procedure described for compound 6 to afford compound **7** (28.8 mg, 96%) as a white solid. ^1^H-NMR (400 MHz, D_2_O): δ 5.86 (m, 1 H), 5.19–4.80 (m, 2 H), 4.08 (t, *J* = 6.0 Hz, 1 H), 3.94 (t, *J* = 5.6 Hz, 2 H), 3.72 (m, 2 H), 1.95 (m, 2 H), 1.69 (m, 2 H), 1.50 (m, 2 H) ppm; ^13^C-NMR (100 MHz, D_2_O): δ 173.2, 159.0, 135.4, 115.1, 63.1, 53.7, 42.1, 29.7, 28.0, 21.0 ppm; HRMS-ESI (*m/z*): [M+Na]^+^ calcd for C_15_H_26_N_2_O_6_ 353.1689, found 353.1654.

### Procedure for the synthesis of 8

#### (S)-2-((tert-Butoxycarbonyl)amino)-6-(pent-4-enamido)hexanoic acid (8a)

Compound **8a** (212 mg, 97%) was obtained as an off-white foam from 2,5-dioxopyrrolidin-1-yl pent-4-enoate [96] (132 mg, 0.67 mmol) by following the procedure described for compound 2b. ^1^H-NMR (400 MHz, CDCl_3_): δ 9.56 (s, br, 1 H), 6.20 (s, br, 1 H), 5.78 (m, 1 H), 5.34 (m, 1 H), 5.06–4.97 (m, 2 H), 4.25 (m, br, 1 H), 4.46 (s, br, 1 H), 3.23 (m, 2 H), 2.34 (m, 2 H), 2.27 (m, 2 H), 1.82–1.66 (m, br, 2 H), 1.59–1.35 (m, 13 H) ppm; ^13^C-NMR (100 MHz, CDCl_3_): δ 175.4, 173.5, 156.0, 137.0, 115.8, 80.1, 53.2, 39.3, 35.8, 32.3, 29.8, 28.9, 28.4, 22.5 ppm; HRMS-ESI (*m/z*): [M+K]^+^ calcd for C_16_H_28_N_2_O_5_ 367.1635, found 367.1625.

#### (S)-2-Amino-6-(pent-4-enamido)hexanoic acid HCl salt (8)

Deprotection of **8a** (175 mg, 0.54 mmol) was performed by following the procedure described for compound 2 to afford compound **8** (142 mg, 99%) as a white solid. ^1^H-NMR (400 MHz, DMSO-d6): δ 8.45 (s, br, 3 H), 7.93 (m, br, 1 H), 5.77 (m, 1 H), 5.02–4.92 (m, 2 H), 3.95 (m, br, 1 H), 3.81 (s, br, 1 H), 3.00 (m, 2 H), 2.25–2.14 (m, 4 H), 1.79 (m, br, 2 H), 1.40–1.29 (m, br, 4 H) ppm; ^13^C-NMR (100 MHz, DMSO-d6): δ 171.3, 171.0, 137.8, 115.0, 51.9, 38.0, 34.5, 29.6, 29.3, 28.6, 21.7 ppm; HRMS-ESI (*m/z*): [M+H]^+^ calcd for C_11_H_20_N_2_O_3_ 229.1547, found 229.1545.

### Procedure for the synthesis of 9

#### (S)-6-(3-Allylureido)-2-((tert-butoxycarbonyl)amino)hexanoic acid (9a)

Allyl isocyanate (100 µL, 1.13 mmol) was dissolved in dry DMF (2 mL) and the solution was chilled to 0°C before adding Boc-Lys-OH (334 mg, 1.36 mmol) and DMAP (166 mg, 1.36 mmol). The reaction was heated at 70°C overnight. After cooling to room temperature, the mixture was diluted in water (6 mL) and extracted with EtOAc (3×6 mL). The combined organic layers were washed with water (3×15 mL) and brine (8 mL). The resulting organic layer was dried over Na_2_SO_4_, filtered and concentrated in vacuo to dryness to furnish **9a** (213 mg, 57% yield) as an off-white foam. ^1^H-NMR (400 MHz, CDCl_3_): δ 8.78 (s, b, 1 H), 6.19 (s, br, 0.5 H), 5.87–5.77 (m, 1 H), 5.46 (d, *J* = 7.2 Hz, 1 H), 5.20–5.09 (m, 2 H), 4.25 (m, br, 1 H), 4.11 (m, br, 0.5 H), 3.77 (m, br, 2 H), 3.12 (m, br, 2 H), 1.80–1.68 (m, 2 H), 1.58–1.43 (m, 13 H) ppm; ^13^C-NMR (100 MHz, CDCl_3_): δ 176.0, 159.7, 156.0, 135.2, 116.1, 80.2, 53.5, 43.2, 40.3, 32.3, 29.4, 28.6, 22.5 ppm; HRMS-ESI (*m/z*): [M+Na]^+^ calcd for C_15_H_27_N_3_O_5_ 352.1848, found 352.1845.

#### (S)-6-(3-Allylureido)-2-aminohexanoic acid TFA salt (9)

Deprotection of **9a** (53.7 mg, 0.16 mmol) was performed by following the procedure described for compound 6 to afford compound **9** (50 mg, 94%) as a white solid. ^1^H-NMR (400 MHz, D_2_O): δ 5.62–5.55 (m, 1 H), 4.93–4.84 (m, 2 H), 3.81 (t, *J* = 6.0 Hz, 1 H), 3.45 (m, 2 H), 2.85 (m, 2 H), 1.72–1.58 (m, 2 H), 1.30–1.09 (m, 4 H) ppm; ^13^C-NMR (100 MHz, D_2_O): δ 172.0, 160.5, 135.1, 114.6, 52.7, 42.0, 39.4, 29.4, 28.7, 21.5 ppm; HRMS-ESI (*m/z*): [M+H]^+^ calcd for C_10_H_19_N_3_O_3_ 230.15, found 230.14.

### Procedure for the synthesis of 10

#### 5-(Dimethylamino)-N-(2-sulfanylethyl)naphthalene-1-Sulfonamide (dansyl-thiol, 10)

A solution of dansyl chloride (150 mg, 0.56 mmol) and TEA (194 µL, 1.39 mmol) in dry DCM (0.8 mL) was cooled to 0°C and added dropwise into an ice-cold solution of cysteamine (86 mg, 1.11 mmol) in dry DCM (1 mL). The reaction was allowed to stir at room temperature for 3 h, was concentrated, and the product was purified on silica gel, eluting with 97∶2∶1 DCM/Hexanes/TEA to furnish **10** (51.4 mg, 30%) as a yellow film. Characterization data matched with literature [97].

### Myoglobin Expression in *E. coli*

Plasmids, pMyo4TAGpylT and pBKpylS, were co-transformed into *E. coli* Top10 cells as previously described [98] and selected with 25 µg/mL tetracycline and 50 µg/mL kanamycin. A single colony was used to inoculate 2 mL LB medium containing the same antibiotics and grown overnight. Next, 500 µL of culture was used to seed 50 mL of LB culture containing 1 mM of the corresponding UAAs and antibiotics. The pH was adjusted to 7 with 10 M NaOH immediately before inoculation. Cells were then cultivated to OD_600_ = 0.6 and 100 µL of 20% arabinose solution was supplemented to induce arabinose promoter driven expression. The cells were cultivated at 37°C shaker overnight and harvested by centrifugation at 3000 g in standard 50 mL conical tubes. Lysis of the cell was conducted by re-suspending the cell pellets with standard Ni-NTA phosphate lysis buffer with lysozyme and 0.1% Triton X-100. After 1 hour of incubation at 4°C, cells were sonicated on ice to release the soluble portion and debris was removed by centrifugation. The cleared lysates were incubated with 100 µL of Qiagen Ni-NTA agarose slurry at 4°C to bind His-tagged myoglobin. The mixture was then centrifuged at 1000 g for 5 min and agarose beads were collected and transferred to microcentrifugator filter columns. Beads were washed three times with 400 µL Ni-NTA lysis buffer and one time with 400 µL Ni-NTA wash buffer. The protein was eluted with 400 µL of elution buffer. Eluted sample was mixed with SDS loading buffer, heated at 95°C for 5 min and loaded onto 10% SDS-PAGE gel with 1.5 mm thickness and ran at 150 V for 50 min. The gel was stained overnight with Coomassie blue solution (0.1% Coomassie blue, 10% acetic acid, 40% ethanol), then de-stained (10% acetic acid, 40% ethanol) and analyzed (Figure S1). The protein was dialyzed in 1 L of 20 mM ammonium acetate buffer for mass spectrum analysis.

### sfGFP Expression in *E. coli*

The plasmid pMyo4TAGpylT [98] was modified by replacing the myoglobin coding sequence with the sfGFP gene with an amber stop codon mutation placed on Y151 position located on the outer beta sheet domain. The co-transformation was the same as described above but condensed culture protocol was used to maximize UAA yields. The 2 mL of overnight culture was scaled-up to 400 mL culture in 2×1 L Erlenmeyer flask and grown to OD_600_ = 0.6. Cells were harvested in 4×50 mL conical tubes and re-suspended in 50 mL of LB medium containing 1 mM of the corresponding UAA, antibiotics, and 0.1% arabinose. Cells were re-suspended by incubating in a rotary shaker at 37°C for 10 min and collected in a 250 mL Erlenmeyer flask. The cells were induced for 4 h and harvested by centrifugation. Cell pellets were first suspended by 3.6 mL of 50 mM Tris-HCl pH 8.0, supplemented with 2.4 mL of 4 M ammonium sulfate and extracted by three-phase partitioning method [99] with 6 mL of *t*-butanol and vigorous shaking. The aqueous bottom layer containing sfGFP was removed and dialyzed against 1 L Ni-NTA lysis buffer for 1–2 h to remove most of the ammonium sulfate. The dialyzed samples were filtered through 0.45 µm disc filter before loading into Ni-NTA gravity column containing 0.5 mL bed volume. The proteins were bound and washed with 12 mL bed volume of lysis buffer, 6 mL bed volume of wash buffer containing 50 mM imidazole and eluted with Ni-NTA elution buffer. Samples were analyzed by SDS-PAGE, dialyzed against PBS pH 7.4 for subsequent labeling reaction and then dialyzed against 20 mM ammonium acetate for mass spectrum analysis.

### Protein MS Analysis

Protein MS was measured at the Genomics and Proteomics Core Laboratories, University of Pittsburgh. The protein solution was adjusted to 5 pmol/µL in 80% acetonitrile and 0.1% aqueous formic acid. The sample was injected into a Bruker micrOTOF with an Ultimate 3000 HPLC. The results were deconvoluted to calculate the molecular weight using HyStar.

### Thiol-ene Reactions with sfGFP

A reaction buffer containing 30 µL of 1 M TrisHCl pH 6.8 (120 mM), 50 µL of 10% SDS (2%), 50 µL of 10 mM TCEP (2 mM), and 120 µL of water was made. In another eppendorf tube, a solution of 10 mM photoinitiator I2959 containing 50% DMSO in water was prepared. Then 62.5 µL of I2959 were added to 250 µL of reaction buffer just before labeling. Next, 2.5 µL of reaction buffer/photoinitiator mix were added to 20 µL of sfGFP (2400 ng/µL) and incubated at room temperature for 10 min. Dansyl-thiol (**10**) 50X substrate solution was prepared with 10 µL of 100 mM TCEP (10 mM), 20 µL of 25 mM dansyl-thiol in DMSO and 70 µL of deionized water. Next, 16.8 µL of this solution was added to the reaction mixture and incubated at room temperature for another 10 min. Subsequently, 250 µL of 1X SDS loading buffer containing 2-mercaptoethanol and additional 50 µL of 100 mM DTT were prepared for stopping the reaction. Then 12 µL of the reaction samples were aliquoted into 200 µL PCR tubes. Samples were placed on a standard UV transilluminator at 365 nm for 5 min and the reaction was stopped by adding 12 µL of 1X SDS loading buffer to the mixture and heated at 95°C for 5 min. Next, 5 µL of the samples were loaded onto a 10% SDS-PAGE gel with 1.5 mm thickness and ran at 150 V for 50 min. After electroporation, gels were rinsed briefly with deionized water and imaged. Gels were stained with coomassie blue and scanned to visualize the protein bands.

For protein heterodimer formation, the thiol-ene conjugation was carried out with a denatured and reduced lysozyme solution. A reaction buffer containing 120 µL of 1 M TrisHCl pH 6.8, 200 µL of 10% SDS, and 1 mL of 10 mM TCEP was prepared. In 1.32 mL of the reaction buffer, 19 mg of lysozyme were dissolved (1 mM). The solution was sealed in a 2 mL micro-centrifugation tube with a rubber septum and purged with nitrogen for 30 min. The tube containing the protein solution was then heated at 75°C for 30 min. Photo-initiator I2959 was diluted to 10 mM in a solution of 50% DMSO in water. sfGFP solutions were adjusted to a concentration of 23 µM (650 ng/µL) and 20 µL of this solution was mixed with 2 µL of reduced lysozyme and 0.5 µL of I2959 in the dark. The PCR tube containing this mixture was then placed on a standard UV transilluminator at 365 nm for 10 min. A solution containing 120 µL of 1 M Tris-HCl pH 6.8, 20 µL of 10% SDS, 10 µL of 1 M TCEP and 200 µL of glycerol was prepared and 20 µL of it were immediately added to the reaction solution after irradiation. 15 µL of the resulted sample was loaded onto native or 10% SDS-PAGE gel following standard procedures.

### Circular dichroism analysis of lysozyme

CD experiments were performed on an Olis Circular Dichroism Spectrophotometer using 0.1 cm quartz cuvettes. A solution containing 19 mg of lysozyme in 1.32 mL 10 mM phosphate buffer pH 7.4 was prepared. SDS was added to the final concentration of 2%. The lysozyme concentration was diluted to 20 uM for the CD experiment, and CD spectra were collected from 195 to 260 nm in 1 nm increments with an integration time of 5 s and a bandwidth of 2 nm. Increased intensity in the far-UV spectrum with the addition of SDS (Figure S2) is in agreement with previous observations [100].

## Supporting Information

Figure S1 **SDS-PAGE analysis for the incorporation of alkene-bearing lysines 1-9 into myoglobin.** –AA: no UAA was supplemented; +AA: positive control UAA (1 mM); 1-9: myoglobin expression in the presence of the corresponding UAA (1 mM).
(TIF)

Figure S2 **Circular dichroism (CD) spectrum of lysozyme with and without SDS treatment.** Blue: lysozyme with no SDS; Red: lysozyme with 2% SDS.
(TIF)

Scheme S1 **Synthesis of alkene-bearing lysines 2–6.**
(TIF)

Scheme S2 **Synthesis of alkene-bearing lysine 7.**
(TIF)

Scheme S3 **Synthesis of alkene-bearing lysine 8.**
(TIF)

Scheme S4 **Synthesis of alkene-bearing lysine 9.**
(TIF)

Scheme S5 **Synthesis of dansyl-thiol, 10.**
(TIF)

## References

[1] Stephanopoulos N, Francis MB (2011) Choosing an effective protein bioconjugation strategy. Nat Chem Biol 7: 876–884. 22086289 10.1038/nchembio.720

[2] Tsien RY (2009) Constructing and Exploiting the Fluorescent Protein Paintbox (Nobel Lecture). Angew Chem Int Ed Engl 48: 5612–5626. 19565590 10.1002/anie.200901916

[3] Giepmans BN, Adams SR, Ellisman MH, Tsien RY (2006) The fluorescent toolbox for assessing protein location and function. Science 312: 217–224. 16614209 10.1126/science.1124618

[4] Andresen M, Schmitz-Salue R, Jakobs S (2004) Short tetracysteine tags to beta-tubulin demonstrate the significance of small labels for live cell imaging. Mol Biol Cell 15: 5616–5622. 15469986 10.1091/mbc.E04-06-0454 PMC532040

[5] Hinner MJ, Johnsson K (2010) How to obtain labeled proteins and what to do with them. Curr Opin Biotechnol 21: 766–776. 21030243 10.1016/j.copbio.2010.09.011

[6] Sletten EM, Bertozzi CR (2009) Bioorthogonal chemistry: fishing for selectivity in a sea of functionality. Angew Chem Int Ed Engl 48: 6974–6998. 19714693 10.1002/anie.200900942 PMC2864149

[7] Wan W, Wang Y-S, Liu WR (2012) Genetically encoding bioorthogonal functional groups for site-selective protein labeling. Organic Chem Curr Res 1: 1–7.

[8] Davis L, Chin JW (2012) Designer proteins: applications of genetic code expansion in cell biology. Nat Rev Mol Cell Biol 13: 168–182. 22334143 10.1038/nrm3286

[9] Chalker JM, Bernardes GJL, Davis BG (2011) A “Tag-and-Modify” Approach to Site-Selective Protein Modification. Acc Chem Res 44: 730–741. 21563755 10.1021/ar200056q

[10] Liu CC, Schultz PG (2010) Adding new chemistries to the genetic code. Annu Rev Biochem 79: 413–444. 20307192 10.1146/annurev.biochem.052308.105824

[11] Huang Y, Wan W, Russell WK, Pai PJ, Wang Z, et al (2010) Genetic incorporation of an aliphatic keto-containing amino acid into proteins for their site-specific modifications. Bioorg Med Chem Lett 20: 878–880. 20074948 10.1016/j.bmcl.2009.12.077

[12] Wang L, Zhang Z, Brock A, Schultz PG (2003) Addition of the keto functional group to the genetic code of Escherichia coli. Proc Natl Acad Sci U S A 100: 56–61. 12518054 10.1073/pnas.0234824100 PMC140882

[13] Zhang Z, Smith BA, Wang L, Brock A, Cho C, et al (2003) A new strategy for the site-specific modification of proteins in vivo. Biochemistry 42: 6735–6746. 12779328 10.1021/bi0300231

[14] Zeng H, Xie J, Schultz PG (2006) Genetic introduction of a diketone-containing amino acid into proteins. Bioorg Med Chem Lett 16: 5356–5359. 16934461 10.1016/j.bmcl.2006.07.094

[15] Hao Z, Song Y, Lin S, Yang M, Liang Y, et al (2011) A readily synthesized cyclic pyrrolysine analogue for site-specific protein “click” labeling. Chem Commun 47: 4502–4504. 10.1039/c1cc00024a 21387054

[16] Eger S, Castrec B, Hübscher U, Scheffner M, Rubini M, et al (2011) Generation of a mono-ubiquitinated PCNA mimic by click chemistry. Chembiochem 12: 2807–2812. 22052741 10.1002/cbic.201100444

[17] Nguyen DP, Lusic H, Neumann H, Kapadnis PB, Deiters A, et al (2009) Genetic encoding and labeling of aliphatic azides and alkynes in recombinant proteins via a pyrrolysyl-tRNA Synthetase/tRNA(CUA) pair and click chemistry. J Am Chem Soc 131: 8720–8721. 19514718 10.1021/ja900553w

[18] Tsao ML, Tian F, Schultz PG (2005) Selective Staudinger modification of proteins containing p-azidophenylalanine. Chembiochem 6: 2147–2149. 16317766 10.1002/cbic.200500314

[19] Deiters A, Cropp TA, Summerer D, Mukherji M, Schultz PG (2004) Site-specific PEGylation of proteins containing unnatural amino acids. Bioorg Med Chem Lett 14: 5743–5745. 15501033 10.1016/j.bmcl.2004.09.059

[20] Chin JW, Santoro SW, Martin AB, King DS, Wang L, et al (2002) Addition of p-azido-L-phenylalanine to the genetic code of Escherichia coli. J Am Chem Soc 124: 9026–9027. 12148987 10.1021/ja027007w

[21] Lee YJ, Wu B, Raymond JE, Zeng Y, Fang X, et al (2013) A genetically encoded acrylamide functionality. ACS Chem Biol 8: 1664–1670. 23735044 10.1021/cb400267m PMC3746000

[22] Li Y, Yang M, Huang Y, Song X, Liu L, et al (2012) Genetically encoded alkenyl-pyrrolysine analogues for thiol-ene reaction mediated site-specific protein labeling. Chem Sci 3: 2766–2770.

[23] Lang K, Davis L, Torres-Kolbus J, Chou C, Deiters A, et al (2012) Genetically encoded norbornene directs site-specific cellular protein labelling via a rapid bioorthogonal reaction. Nat Chem 4: 298–304. 22437715 10.1038/nchem.1250 PMC3758886

[24] Lang K, Davis L, Wallace S, Mahesh M, Cox DJ, et al (2012) Genetic Encoding of Bicyclononynes and trans-Cyclooctenes for Site-Specific Protein Labeling in Vitro and in Live Mammalian Cells via Rapid Fluorogenic Diels–Alder Reactions. J Am Chem Soc 134: 10317–10320. 22694658 10.1021/ja302832g PMC3687367

[25] Yu Z, Pan Y, Wang Z, Wang J, Lin Q (2012) Genetically encoded cyclopropene directs rapid, photoclick-chemistry-mediated protein labeling in mammalian cells. Angew Chem Int Ed Engl 51: 10600–10604. 22997015 10.1002/anie.201205352 PMC3517012

[26] Ai HW, Shen W, Brustad E, Schultz PG (2010) Genetically encoded alkenes in yeast. Angew Chem Int Ed Engl 49: 935–937. 20025009 10.1002/anie.200905590

[27] Song W, Wang Y, Yu Z, Vera CI, Qu J, et al (2010) A metabolic alkene reporter for spatiotemporally controlled imaging of newly synthesized proteins in Mammalian cells. ACS Chem Biol 5: 875–885. 20666508 10.1021/cb100193h PMC2942984

[28] Song W, Wang Y, Qu J, Lin Q (2008) Selective functionalization of a genetically encoded alkene-containing protein via “photoclick chemistry” in bacterial cells. J Am Chem Soc 130: 9654–9655. 18593155 10.1021/ja803598e

[29] Li Y, Pan M, Huang Y, Guo Q (2013) Thiol-yne radical reaction mediated site-specific protein labeling via genetic incorporation of an alkynyl-L-lysine analogue. Org Biomol Chem 11: 2624–2629. 23450369 10.1039/c3ob27116a

[30] Plass T, Milles S, Koehler C, Szymański J, Mueller R, et al (2012) Amino Acids for Diels–Alder Reactions in Living Cells. Angew Chem Int Ed Engl 51: 4166–4170. 22473599 10.1002/anie.201108231

[31] Plass T, Milles S, Koehler C, Schultz C, Lemke EA (2011) Genetically encoded copper-free click chemistry. Angew Chem Int Ed Engl 50: 3878–3881. 21433234 10.1002/anie.201008178 PMC3210829

[32] Deiters A, Schultz PG (2005) In vivo incorporation of an alkyne into proteins in Escherichia coli. Bioorg Med Chem Lett 15: 1521–1524. 15713420 10.1016/j.bmcl.2004.12.065

[33] Seitchik JL, Peeler JC, Taylor MT, Blackman ML, Rhoads TW, et al (2012) Genetically Encoded Tetrazine Amino Acid Directs Rapid Site-Specific in Vivo Bioorthogonal Ligation with trans-Cyclooctenes. J Am Chem Soc 134: 2898–2901. 22283158 10.1021/ja2109745 PMC3369569

[34] Kodama K, Fukuzawa S, Nakayama H, Kigawa T, Sakamoto K, et al (2006) Regioselective carbon-carbon bond formation in proteins with palladium catalysis; new protein chemistry by organometallic chemistry. Chembiochem 7: 134–139. 16307466 10.1002/cbic.200500290

[35] Kwon I, Wang P, Tirrell DA (2006) Design of a bacterial host for site-specific incorporation of p-bromophenylalanine into recombinant proteins. J Am Chem Soc 128: 11778–11783. 16953616 10.1021/ja0626281

[36] Brustad E, Bushey ML, Lee JW, Groff D, Liu W, et al (2008) A genetically encoded boronate-containing amino acid. Angew Chem Int Ed Engl 47: 8220–8223. 18816552 10.1002/anie.200803240 PMC2873848

[37] Bar-Or R, Rael LT, Bar-Or D (2008) Dehydroalanine derived from cysteine is a common post-translational modification in human serum albumin. Rapid Commun Mass Spectrom 22: 711–716. 18265430 10.1002/rcm.3421

[38] Wickner RB (1969) Dehydroalanine in Histidine Ammonia Lyase. J Biol Chem 244: 6550–6552. 5354969

[39] Chalker JM, Lin YA, Boutureira O, Davis BG (2009) Enabling olefin metathesis on proteins: chemical methods for installation of S-allyl cysteine. Chem Commun (Camb): 3714–3716. 10.1039/b908004j 19557258

[40] Garber KCA, Carlson EE (2013) Thiol-ene Enabled Detection of Thiophosphorylated Kinase Substrates. ACS Chem Biol 8: 1671–1676. 23668631 10.1021/cb400184v PMC3745782

[41] Weinrich D, Lin PC, Jonkheijm P, Nguyen UT, Schröder H, et al (2010) Oriented immobilization of farnesylated proteins by the thiol-ene reaction. Angew Chem Int Ed Engl 49: 1252–1257. 20069617 10.1002/anie.200906190

[42] Trang VH, Valkevich EM, Minami S, Chen Y-C, Ge Y, et al (2012) Nonenzymatic Polymerization of Ubiquitin: Single-Step Synthesis and Isolation of Discrete Ubiquitin Oligomers. Angew Chem Int Ed Engl 51: 13085–13088. 23161800 10.1002/anie.201207171 PMC4083817

[43] Valkevich EM, Guenette RG, Sanchez NA, Chen YC, Ge Y, et al (2012) Forging isopeptide bonds using thiol-ene chemistry: site-specific coupling of ubiquitin molecules for studying the activity of isopeptidases. J Am Chem Soc 134: 6916–6919. 22497214 10.1021/ja300500a PMC4373626

[44] Jonkheijm P, Weinrich D, Köhn M, Engelkamp H, Christianen PC, et al (2008) Photochemical surface patterning by the thiol-ene reaction. Angew Chem Int Ed Engl 47: 4421–4424. 18428169 10.1002/anie.200800101

[45] Chen Y-X, Triola G, Waldmann H (2011) Bioorthogonal Chemistry for Site-Specific Labeling and Surface Immobilization of Proteins. Acc Chem Res 44: 762–773. 21648407 10.1021/ar200046h

[46] Lang K, Chin JW (2014) Bioorthogonal Reactions for Labeling Proteins. ACS Chem Biol 9: 16–20. 24432752 10.1021/cb4009292

[47] Lim RK, Lin Q (2010) Bioorthogonal chemistry: recent progress and future directions. Chem Commun (Camb) 46: 1589–1600. 20177591 10.1039/b925931g PMC2914230

[48] Klemm JD, Schreiber SL, Crabtree GR (1998) Dimerization as a regulatory mechanism in signal transduction. Annu Rev Immunol 16: 569–592. 9597142 10.1146/annurev.immunol.16.1.569

[49] Funnell AW, Crossley M (2012) Homo- and Heterodimerization in Transcriptional Regulation. In: Matthews JM, editor. Protein Dimerization and Oligomerization in Biology: Springer New York. pp. 105–121. 10.1007/978-1-4614-3229-6_7 22949114

[50] Walker JR, Corpina RA, Goldberg J (2001) Structure of the Ku heterodimer bound to DNA and its implications for double-strand break repair. Nature 412: 607–614. 11493912 10.1038/35088000

[51] McGinty RK, Köhn M, Chatterjee C, Chiang KP, Pratt MR, et al (2009) Structure–Activity Analysis of Semisynthetic Nucleosomes: Mechanistic Insights into the Stimulation of Dot1L by Ubiquitylated Histone H2B. ACS Chem Biol 4: 958–968. 19799466 10.1021/cb9002255 PMC2785503

[52] Xiao J, Tolbert TJ (2009) Synthesis of N-Terminally Linked Protein Dimers and Trimers by a Combined Native Chemical Ligation-CuAAC Click Chemistry Strategy. Org Lett 11: 4144–4147. 19705863 10.1021/ol9016468

[53] Dawson PE, Kent SBH (2000) Synthesis of native proteins by chemical ligation. Annu Rev Biochem 69: 923–960. 10966479 10.1146/annurev.biochem.69.1.923

[54] Dawson PE (2011) Native Chemical Ligation Combined with Desulfurization and Deselenization: A General Strategy for Chemical Protein Synthesis. Isr J Chem 51: 862–867.

[55] Blaschke UK, Silberstein J, Muir TW (2000) Protein engineering by expressed protein ligation. In: Thorner J, Emr SD, Abelson JN, editors. Methods in Enzymology. Academic Press. pp. 478–496. 10.1016/s0076-6879(00)28414-0 11075362

[56] Levary DA, Parthasarathy R, Boder ET, Ackerman ME (2011) Protein-protein fusion catalyzed by sortase A. PLoS ONE. 6: e18342. 10.1371/journal.pone.0018342 PMC3071835 21494692

[57] Li X, Fekner T, Ottesen JJ, Chan MK (2009) A pyrrolysine analogue for site-specific protein ubiquitination. Angew Chem Int Ed Engl 48: 9184–9187. 19882608 10.1002/anie.200904472

[58] Virdee S, Kapadnis PB, Elliott T, Lang K, Madrzak J, et al (2011) Traceless and site-specific ubiquitination of recombinant proteins. J Am Chem Soc 133: 10708–10711. 21710965 10.1021/ja202799r PMC3135006

[59] Eger S, Scheffner M, Marx A, Rubini M (2010) Synthesis of defined ubiquitin dimers. J Am Chem Soc 132: 16337–16339. 21033666 10.1021/ja1072838

[60] Sommer S, Weikart ND, Brockmeyer A, Janning P, Mootz HD (2011) Expanded click conjugation of recombinant proteins with ubiquitin-like modifiers reveals altered substrate preference of SUMO2-modified Ubc9. Angew Chem Int Ed Engl 50: 9888–9892. 21898723 10.1002/anie.201102531

[61] Bundy BC, Swartz JR (2010) Site-Specific Incorporation of p-Propargyloxyphenylalanine in a Cell-Free Environment for Direct Protein−Protein Click Conjugation. Bioconj Chem 21: 255–263. 10.1021/bc9002844 20099875

[62] Hutchins BM, Kazane SA, Staflin K, Forsyth JS, Felding-Habermann B, et al (2011) Selective Formation of Covalent Protein Heterodimers with an Unnatural Amino Acid. Chem Biol 18: 299–303. 21439474 10.1016/j.chembiol.2011.01.006 PMC3694407

[63] Hudak JE, Barfield RM, de Hart GW, Grob P, Nogales E, et al (2012) Synthesis of heterobifunctional protein fusions using copper-free click chemistry and the aldehyde tag. Angew Chem Int Ed Engl 51: 4161–4165. 22407566 10.1002/anie.201108130 PMC3379715

[64] Hoyle CE, Bowman CN (2010) Thiol-ene click chemistry. Angew Chem Int Ed Engl 49: 1540–1573. 20166107 10.1002/anie.200903924

[65] Northrop BH, Coffey RN (2012) Thiol–Ene Click Chemistry: Computational and Kinetic Analysis of the Influence of Alkene Functionality. J Am Chem Soc 134: 13804–13817. 22853003 10.1021/ja305441d

[66] DeForest CA, Anseth KS (2012) Photoreversible Patterning of Biomolecules within Click-Based Hydrogels. Angew Chem Int Ed Engl 51: 1816–1819. 22162285 10.1002/anie.201106463 PMC3430005

[67] Aimetti AA, Machen AJ, Anseth KS (2009) Poly(ethylene glycol) hydrogels formed by thiol-ene photopolymerization for enzyme-responsive protein delivery. Biomaterials 30: 6048–6054. 19674784 10.1016/j.biomaterials.2009.07.043 PMC2761537

[68] Gupta N, Lin BF, Campos LM, Dimitriou MD, Hikita ST, et al (2010) A versatile approach to high-throughput microarrays using thiol-ene chemistry. Nat Chem 2: 138–145. 21124405 10.1038/nchem.478

[69] Chan JW, Yu B, Hoyle CE, Lowe AB (2008) Convergent synthesis of 3-arm star polymers from RAFT-prepared poly(N,N-diethylacrylamide) via a thiol-ene click reaction. Chem Commun (Camb): 4959–4961. 10.1039/b813438c 18931752

[70] Chan JW, Hoyle CE, Lowe AB (2009) Sequential phosphine-catalyzed, nucleophilic thiol-ene/radical-mediated thiol-yne reactions and the facile orthogonal synthesis of polyfunctional materials. J Am Chem Soc 131: 5751–5753. 19341293 10.1021/ja8099135

[71] Lowe AB (2010) Thiol-ene “click” reactions and recent applications in polymer and materials synthesis. Polym Chem 1: 17–36.

[72] Lv Y, Lin Z, Svec F (2012) “Thiol-ene” click chemistry: a facile and versatile route for the functionalization of porous polymer monoliths. Analyst 137: 4114–4118. 22858785 10.1039/c2an35706b PMC3466814

[73] Wojcik F, O'Brien AG, Götze S, Seeberger PH, Hartmann L (2013) Synthesis of Carbohydrate-Functionalised Sequence-Defined Oligo(amidoamine)s by Photochemical Thiol-Ene Coupling in a Continuous Flow Reactor. Chemistry 19: 3090–3098. 23325532 10.1002/chem.201203927

[74] Dondoni A, Marra A (2012) Recent applications of thiol-ene coupling as a click process for glycoconjugation. Chem Soc Rev 41: 573–586. 21792452 10.1039/c1cs15157f

[75] Fekner T, Chan MK (2011) The pyrrolysine translational machinery as a genetic-code expansion tool. Curr Opin Chem Biol 15: 387–391. 21507706 10.1016/j.cbpa.2011.03.007 PMC3487393

[76] Krzycki JA (2005) The direct genetic encoding of pyrrolysine. Curr Opin Microbiol 8: 706–712. 16256420 10.1016/j.mib.2005.10.009

[77] Atkins JF, Gesteland R (2002) Biochemistry. The 22nd amino acid. Science 296: 1409–1410. 12029118 10.1126/science.1073339

[78] Namy O, Zhou Y, Gundllapalli S, Polycarpo CR, Denise A, et al (2007) Adding pyrrolysine to the Escherichia coli genetic code. FEBS Lett 581: 5282–5288. 17967457 10.1016/j.febslet.2007.10.022

[79] Hancock SM, Uprety R, Deiters A, Chin JW (2010) Expanding the genetic code of yeast for incorporation of diverse unnatural amino acids via a pyrrolysyl-tRNA synthetase/tRNA pair. J Am Chem Soc 132: 14819–14824. 20925334 10.1021/ja104609m PMC2956376

[80] Blight SK, Larue RC, Mahapatra A, Longstaff DG, Chang E, et al (2004) Direct charging of tRNA(CUA) with pyrrolysine in vitro and in vivo. Nature 431: 333–335. 15329732 10.1038/nature02895

[81] Greiss S, Chin JW (2011) Expanding the Genetic Code of an Animal. J Am Chem Soc 133: 14196–14199. 21819153 10.1021/ja2054034 PMC3168933

[82] Parrish AR, She X, Xiang Z, Coin I, Shen Z, et al (2012) Expanding the Genetic Code of Caenorhabditis elegans Using Bacterial Aminoacyl-tRNA Synthetase/tRNA Pairs. ACS Chem Biol 7: 1292–1302. 22554080 10.1021/cb200542j PMC3401359

[83] Bianco A, Townsley FM, Greiss S, Lang K, Chin JW (2012) Expanding the genetic code of Drosophila melanogaster. Nat Chem Biol 8: 748–750. 22864544 10.1038/nchembio.1043

[84] Ye S, Riou M, Carvalho S, Paoletti P (2013) Expanding the Genetic Code in Xenopus laevis Oocytes. ChemBioChem 14: 230–235. 23292655 10.1002/cbic.201200515

[85] Yanagisawa T, Ishii R, Fukunaga R, Kobayashi T, Sakamoto K, et al (2008) Multistep engineering of pyrrolysyl-tRNA synthetase to genetically encode N(epsilon)-(o-azidobenzyloxycarbonyl) lysine for site-specific protein modification. Chem Biol 15: 1187–1197. 19022179 10.1016/j.chembiol.2008.10.004

[86] Chou C, Uprety R, Davis L, Chin JW, Deiters A (2011) Genetically encoding an aliphatic diazirine for protein photocrosslinking. Chem Sci 2: 480–483.

[87] Kavran JM, Gundllapalli S, O'Donoghue P, Englert M, Söll D, et al (2007) Structure of pyrrolysyl-tRNA synthetase, an archaeal enzyme for genetic code innovation. Proc Natl Acad Sci U S A 104: 11268–11273. 17592110 10.1073/pnas.0704769104 PMC2040888

[88] Yanagisawa T, Ishii R, Fukunaga R, Kobayashi T, Sakamoto K, et al (2008) Crystallographic studies on multiple conformational states of active-site loops in pyrrolysyl-tRNA synthetase. J Mol Biol 378: 634–652. 18387634 10.1016/j.jmb.2008.02.045

[89] Gattner MJ, Vrabel M, Carell T (2013) Synthesis of ε-N-propionyl-, ε-N-butyryl-, and ε-N-crotonyl-lysine containing histone H3 using the pyrrolysine system. Chem Commun: 379–381. 10.1039/c2cc37836a 23192406

[90] Flügel V, Vrabel M, Schneider S (2014) Structural basis for the site-specific incorporation of lysine derivatives into proteins. PLoS One 9: e96198. 24760130 10.1371/journal.pone.0096198 PMC3997565

[91] Rypniewski WR, Holden HM, Rayment I (1993) Structural consequences of reductive methylation of lysine residues in hen egg white lysozyme: An x-ray analysis at 1.8-.ANG. resolution. Biochemistry 32: 9851–9858. 8373783 10.1021/bi00088a041

[92] Behbehani GR, Saboury AA, Taleshi E (2008) A direct calorimetric determination of denaturation enthalpy for lysozyme in sodium dodecyl sulfate. Colloids Surf B Biointerfaces 61: 224–228. 17889513 10.1016/j.colsurfb.2007.08.007

[93] Michaux C, Pomroy NC, Prive GG (2008) Refolding SDS-denatured proteins by the addition of amphipathic cosolvents. J Mol Biol 375: 1477–1488. 18083190 10.1016/j.jmb.2007.11.026

[94] Durham OZ, Krishnan S, Shipp DA (2012) Polymer Microspheres Prepared by Water-Borne Thiol–Ene Suspension Photopolymerization. ACS Macro Lett 1: 1134–1137. 10.1021/mz300358j 35607182

[95] Tan J, Li C, Zhou J, Yin C, Zhang B, et al (2014) Fast and facile fabrication of porous polymer particles via thiol–ene suspension photopolymerization. RSC Adv 4: 13334–13339.

[96] Arnaud O, Koubeissi A, Ettouati L, Terreux R, Alamé G, et al (2010) Potent and fully noncompetitive peptidomimetic inhibitor of multidrug resistance P-glycoprotein. J Med Chem 53: 6720–6729. 20731360 10.1021/jm100839w

[97] Robinson C, Hartman RF, Rose SD (2008) Emollient, humectant, and fluorescent alpha,beta-unsaturated thiol esters for long-acting skin applications. Bioorg Chem 36: 265–270. 18752827 10.1016/j.bioorg.2008.06.004

[98] Neumann H, Peak-Chew SY, Chin JW (2008) Genetically encoding N(epsilon)-acetyllysine in recombinant proteins. Nat Chem Biol 4: 232–234. 18278036 10.1038/nchembio.73

[99] Jain S, Singh R, Gupta MN (2004) Purification of recombinant green fluorescent protein by three-phase partitioning. J Chromatogr A 1035: 83–86. 15117077 10.1016/j.chroma.2004.01.007

[100] Sureshchandra BR, Rao AGA, Rao MSN (1987) Effect of sodium dodecyl sulfate on the conformation of soybean glycinin. J Agric Food Chem 35: 244–247.

