# Supplementary figures and images for: Synthesis of Non-linear Protein Dimers through a Genetically Encoded Thiol-ene Reaction
Source: PLoS One. 2014 Sep 2;9(9):e105467. doi: 10.1371/journal.pone.0105467 (PMC4152134; doi:10.1371/journal.pone.0105467)

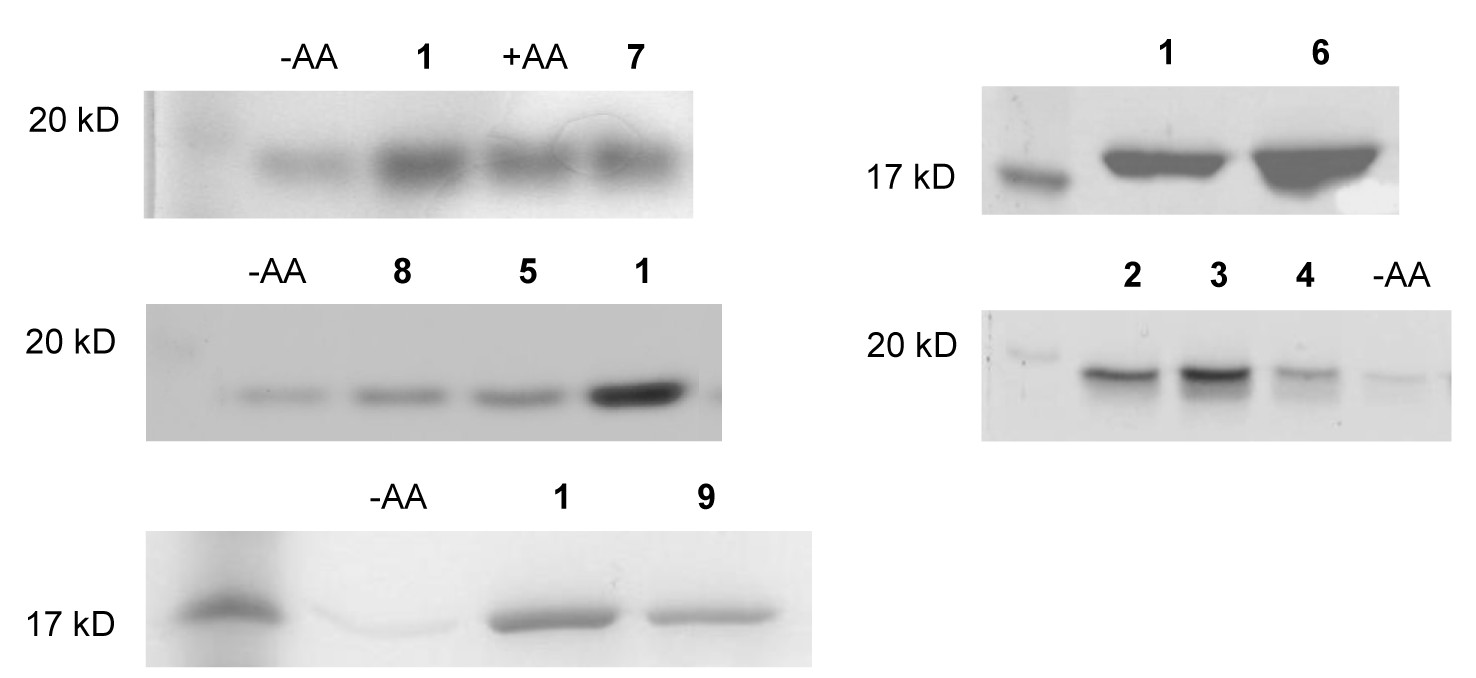

Supplement: Figure S1 — SDS-PAGE analysis for the incorporation of alkene-bearing lysines 1-9 into myoglobin. –AA: no UAA was supplemented; +AA: positive control UAA (1 mM); 1-9: myoglobin expression in the presence of the corresponding UAA (1 mM). (TIF) [file pone.0105467.s001.tif]

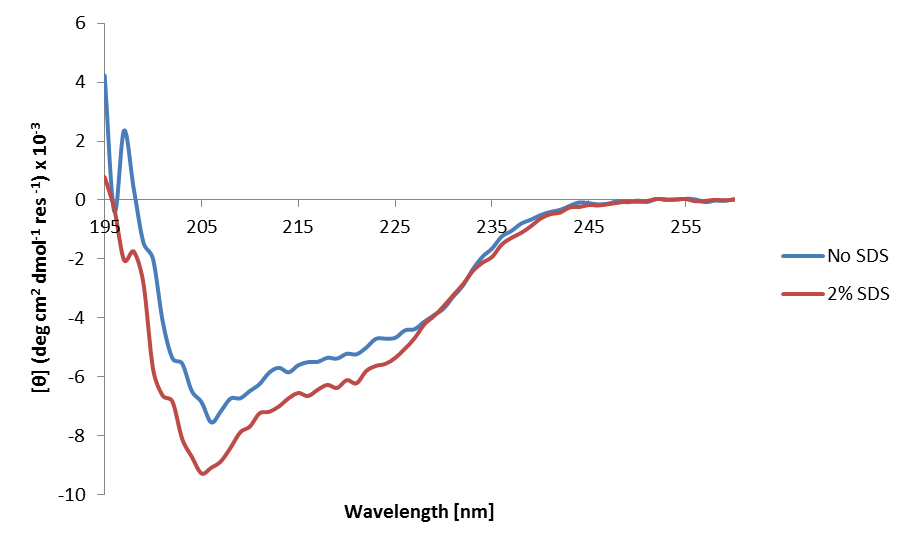

Supplement: Figure S2 — Circular dichroism (CD) spectrum of lysozyme with and without SDS treatment. Blue: lysozyme with no SDS; Red: lysozyme with 2% SDS. (TIF) [file pone.0105467.s002.tif]

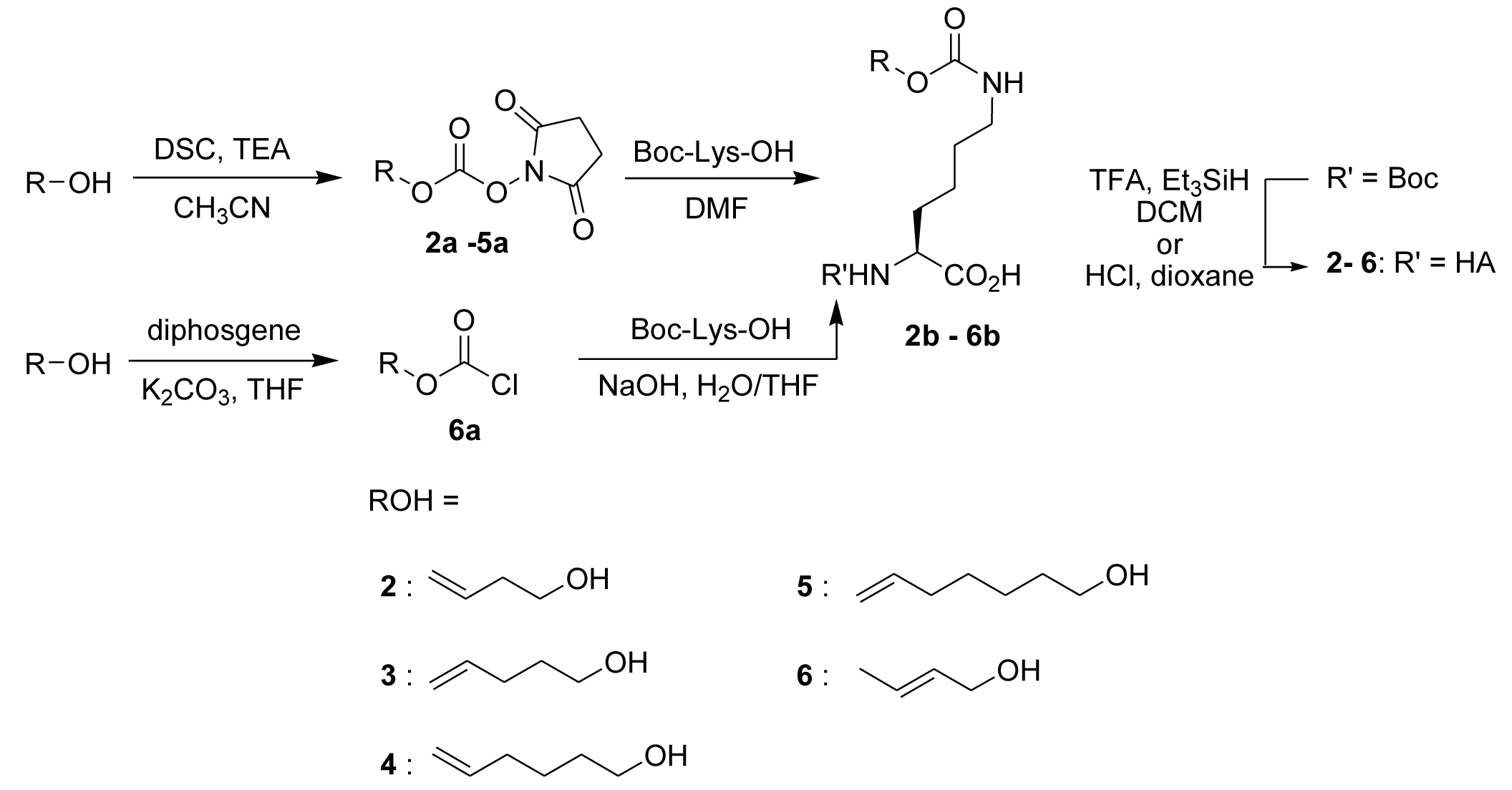

Supplement: Scheme S1 — Synthesis of alkene-bearing lysines 2–6. (TIF) [file pone.0105467.s003.tif]

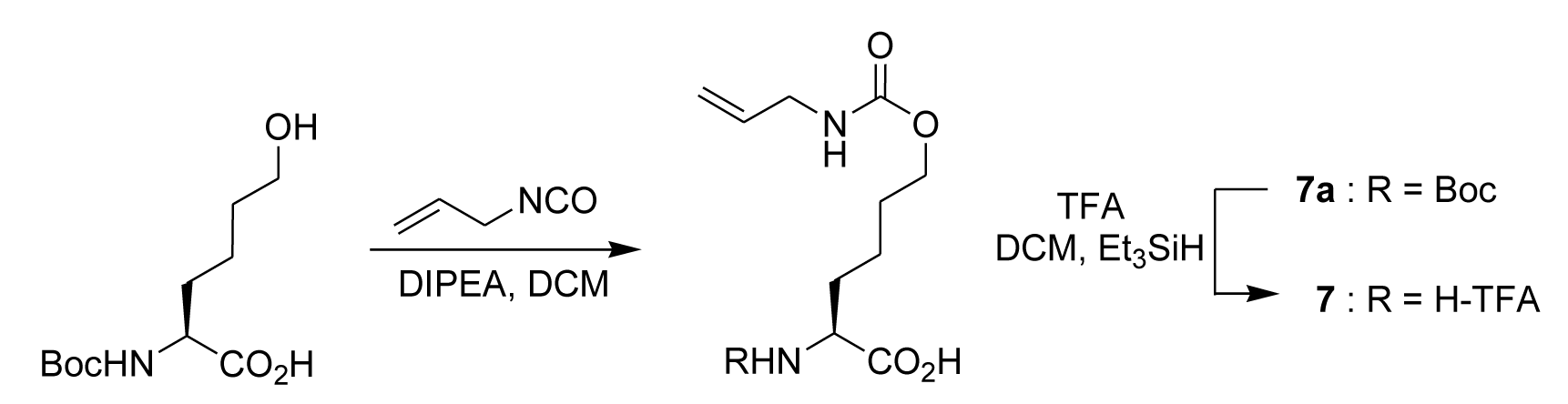

Supplement: Scheme S2 — Synthesis of alkene-bearing lysine 7. (TIF) [file pone.0105467.s004.tif]

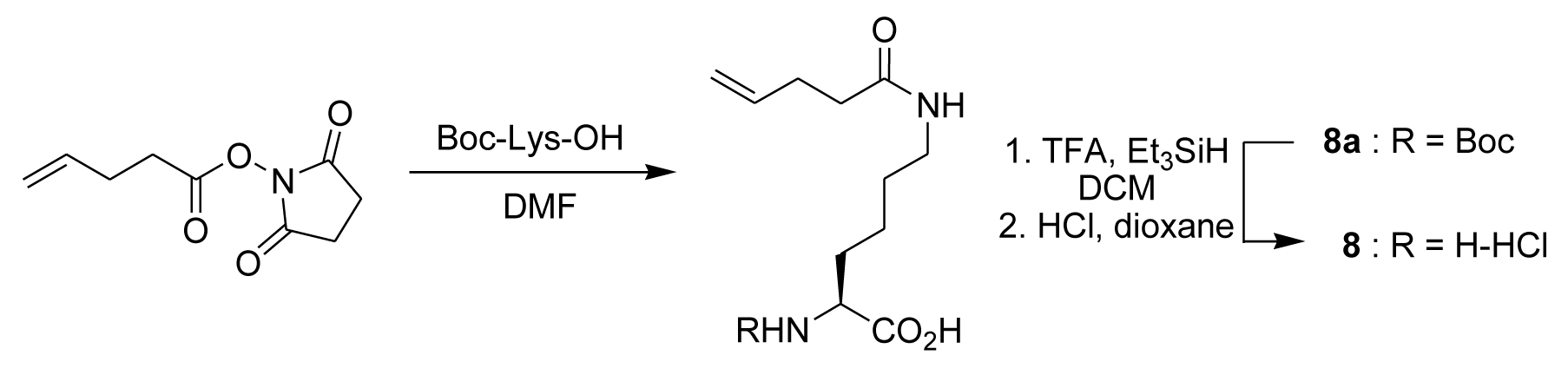

Supplement: Scheme S3 — Synthesis of alkene-bearing lysine 8. (TIF) [file pone.0105467.s005.tif]

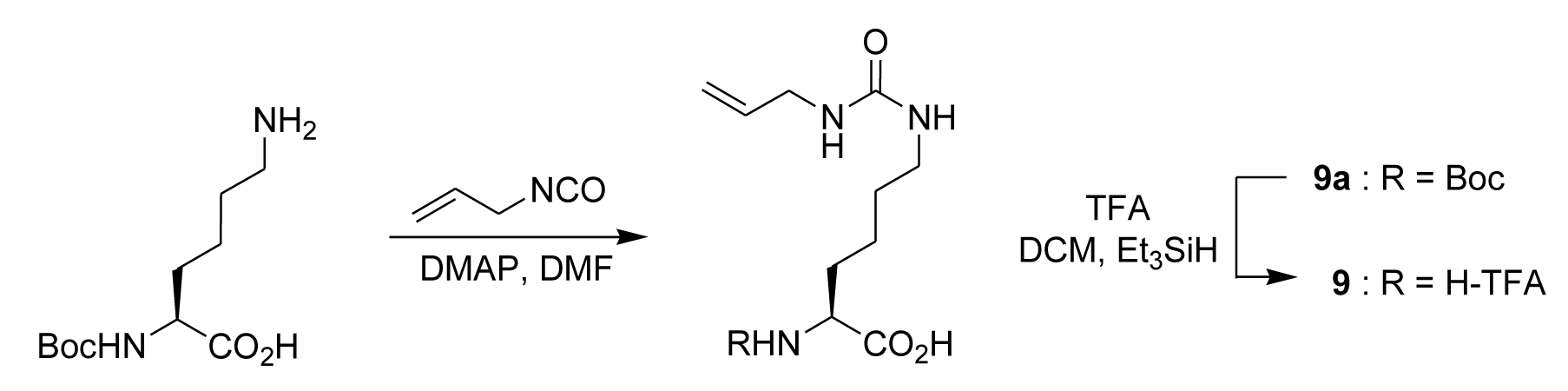

Supplement: Scheme S4 — Synthesis of alkene-bearing lysine 9. (TIF) [file pone.0105467.s006.tif]

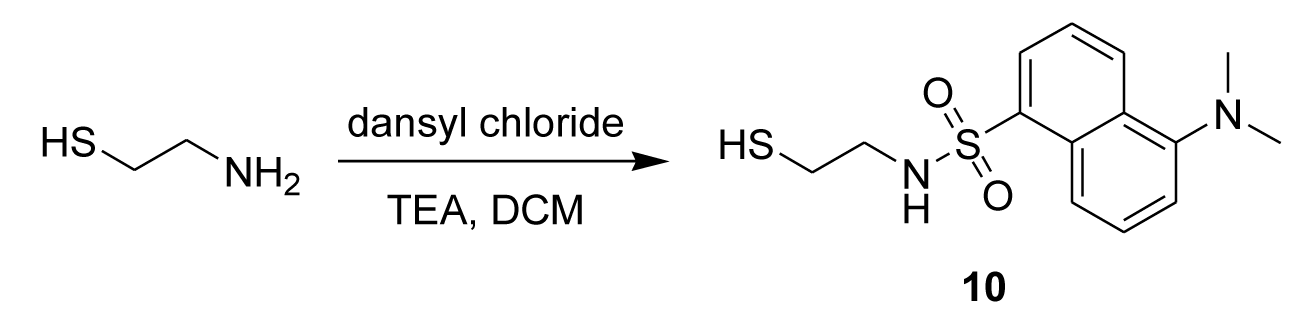

Supplement: Scheme S5 — Synthesis of dansyl-thiol, 10. (TIF) [file pone.0105467.s007.tif]
